# The Potential of Medical Abortion to Reduce Maternal Mortality in Africa: What Benefits for Tanzania and Ethiopia?

**DOI:** 10.1371/journal.pone.0013260

**Published:** 2010-10-11

**Authors:** Rebecca F. Baggaley, Joanna Burgin, Oona M. R. Campbell

**Affiliations:** 1 Department of Infectious Disease Epidemiology, Imperial College London, London, United Kingdom; 2 Medical Development Team, Marie Stopes International, London, United Kingdom; 3 Infectious Disease Epidemiology Unit, Department of Epidemiology and Public Health, London School of Hygiene and Tropical Medicine, London, United Kingdom; University of Swansea, United Kingdom

## Abstract

**Background:**

Unsafe abortion is estimated to account for 13% of maternal mortality globally. Medical abortion is a safe alternative.

**Methods:**

By estimating mortality risks for unsafe and medical abortion and childbirth for Tanzania and Ethiopia, we modelled changes in maternal mortality that are achievable if unsafe abortion were replaced by medical abortion. We selected Ethiopia and Tanzania because of their high maternal mortality ratios (MMRatios) and contrasting situations regarding health care provision and abortion legislation. We focused on misoprostol-only regimens due to the drug's low cost and accessibility. We included the impact of medical abortion on women who would otherwise choose unsafe abortion and on women with unwanted/mistimed pregnancies who would otherwise carry to term.

**Results:**

Thousands of lives could be saved each year in each country by implementing medical abortion using misoprostol (2122 in Tanzania and 2551 in Ethiopia assuming coverage equals family planning services levels: 56% for Tanzania, 31% for Ethiopia). Changes in MMRatios would be less pronounced because the intervention would also affect national birth rates.

**Conclusions:**

This is the first analysis of impact of medical abortion provision which takes into account additional potential users other than those currently using unsafe abortion. Thousands of women's lives could be saved, but this may not be reflected in as substantial changes in MMRatios because of medical abortion's demographic impact. Therefore policy makers must be aware of the inability of some traditional measures of maternal mortality to detect the real benefits offered by such an intervention.

## Introduction

In June 2009, the UN Human Rights Council passed a landmark resolution recognizing preventable maternal mortality and morbidity as a pressing human-rights issue that violates a woman's rights to health, life, education, dignity, and information. Millennium Development Goal (MDG) 5 aims to reduce maternal mortality by three quarters by 2015 [1]. Globally, approximately 67,900 women die each year as a consequence of unsafe abortion (13% of maternal deaths) and around 5.3 million suffer temporary or permanent disability [2], [3]. Provision of safe induced abortion would prevent many of these deaths but 26% of the world's population live in countries where abortion is either completely prohibited or permitted only to save a woman's life [4].

Sub-Saharan Africa has the world's highest maternal mortality ratios (MMRatio) [5]. In this region, 3.9% (range 0.0–23.8%) of maternal deaths are due to induced abortion [6] arising from an estimated 19 million unsafe abortions performed annually [7]. Africa accounts for 25% of all illegal abortions performed worldwide and less than 1% of all legal abortions [8]. The estimated proportion of all pregnancies terminated by induced abortion in Africa is only 15%, the lowest for any continent [9]. This is partly due to strict sanctions against abortion in most African countries, but also from a desire for larger families than the rest of the world [9]. Africa is also one of the most dangerous regions to have an abortion: the ratio of abortion deaths per 100,000 procedures is less than 1/100,000 in developed countries, for developing countries is 330/100,000 and for Africa alone averages 680/100,000 [8].

Medical abortion uses medications in place of traditional surgical interventions to induce abortion [10]. It is a safe procedure, with mortality rates for mifepristone-misoprostol combination regimens that are comparable to spontaneous abortion [12], [13], although if taken late in pregnancy or in higher doses there is a small risk of uterine rupture, hemorrhage and possibly death (mifepristone-misoprostol [11], [12], misoprostol-only [13], [14]).

Mifepristone with misoprostol are the most common drugs used, with success rates of 94–97% [15]. Misoprostol-only is less effective at 73–95% [15]–[26]. Mifepristone use in developing countries has been extremely limited mainly due to licensing restrictions, but also for several other reasons including the high cost of the medication, leading to the exploration of use of a misoprostol-only regimen, since misoprostol is widely used for treating other conditions and is already registered in many countries, including those with restrictive abortion laws. In some settings, it is known to be widely accessed by women and doctors to induce abortion illegally [27]–[30].

Several authors have estimated the potential impact of various interventions on maternal mortality in order to inform decisions on implementation and prioritize funding, many of which evaluate safe abortion strategies [31]–[36]. Harper et al. used a simple model to demonstrate the number of abortion-attributable deaths that could be prevented if misoprostol were made readily available, reporting that up to 68% of such deaths in Africa could be prevented if the majority of women had access [31]. The authors explored the impact of high (80%) and low (20%) access, assuming that 80% of women attempt to access medical abortion during the first-trimester. Here, we extend this approach to explore more closely the different factors influencing a woman's successful use of medical abortion and its potential impact on maternal mortality within sub-Saharan Africa, using Tanzania and Ethiopia as examples.

## Methods

**Figure 1:**
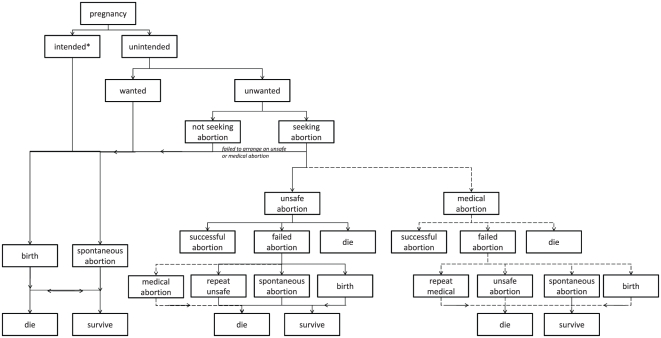
Decision tree of outcomes of pregnancy according to whether medical abortion services are available. Solid lines represent choices available to all women; dotted lines represent choices available to women where medical abortion services are available. * Some intended pregnancies may still result in induced abortion due to fetal abnormalities.

To estimate numbers of deaths averted by introducing medical abortion, we considered a range of possible pregnancy outcomes depending upon whether the pregnancy was intended or wanted and whether a woman sought an abortion. Figure 1 illustrates these outcomes and how they influence each woman's chances of survival. Impact of medical abortion was modeled based on this decision tree. For simplicity, the impact of spontaneous abortion and stillbirth was ignored. Mortality and risk of failure associated with termination during first and second trimesters were specified, as risks of complication and death and failure rates increase later in gestation [11], [37].

Ethiopia and Tanzania were chosen for their high MMRatios, unsafe abortion case-fatality rates and proportions of maternal mortality attributable to unsafe abortion. These countries also provide interesting contrasts as they differ in important areas including abortion legislation (with Ethiopia's law recently liberalized) and provision of health care services (Tanzania has higher access to antenatal care, contraception and health facility treatment for acute respiratory infection (ARI)). Additionally, both countries have recently licensed misoprostol for post-partum hemorrhage [38]. Therefore access to medical abortion using misoprostol is a real possibility because such registration improves availability for obstetric-gynecologic conditions in general. No ethics approval was sought for the study as all data used were taken from published literature.

### Model description

For each country, the number of women currently dying per year from unsafe abortion is 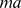: the number of women dying from all maternal causes, 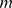, multiplied by the proportion of these women dying from unsafe abortion, 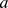. If medical abortion services become available, the number of these deaths that could be averted is limited by coverage, 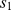, so number of women who would have died from unsafe abortion able to access medical abortion becomes 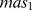.

We assume that all women choose medical abortion over unsafe abortion, that no abortions are performed during the third trimester and that women seek a medical abortion during the same trimester that they would have sought an unsafe abortion. The effectiveness of a medical abortion regimen for first (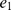) and second (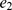) trimesters, the proportion of medical abortions sought during the first trimester (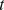) and the mortality risk associated with medical abortion by trimester (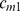 and 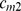) are used to calculate the number of women undergoing successful medical abortion and considered “lives saved” as 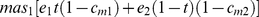.

**Figure 2:**
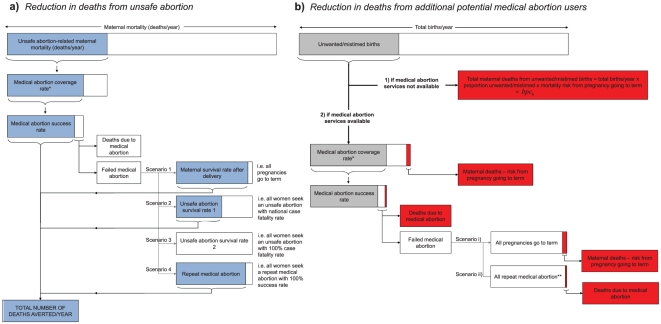
Schematic illustrating method of estimating deaths due to unsafe abortion averted by introducing medical abortion, representing a) the reduction in deaths from unsafe abortions averted and b) the reduction in deaths from the risk associated with pregnancy going to term, for additional potential users of abortion services. For b), deaths averted are the difference between number of deaths from route 1), where medical abortion is not available, and route 2) where they are available. Fractions for each bar are not drawn to scale. * Defined as women who would have accessed unsafe abortion services but preferentially seek medical abortion services, if available. ** Assumes that a repeated medical abortion is 100% effective.

For those women whose medical abortion fails, their survival must also be estimated. Four potential scenarios are considered (Figure 2a): 1) each woman carries her pregnancy to term and experiences the same risk of maternal death as other women giving birth in her country, so the death rate, defined as 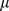, is 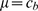, where 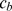 is the MMRatio for her country removing the proportion of maternal deaths due to unsafe abortion i.e. 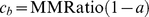; 2) each women undergoes a second (unsafe) abortion and is subject to the population-level case fatality rate for unsafe abortion procedures for that country (assuming that half those failing medical abortion in the first trimester are in the second trimester by the time of the repeat procedure, 
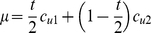
); 3) each woman undergoes unsafe abortion and dies (the assumption being that these women were defined as originally dying from unsafe abortion, if medical abortion was not available: (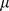 = 100%); and 4) each woman seeks a repeat medical abortion, with the same mortality risk as for their first procedure (same assumption regarding trimesters as Scenario 2, 
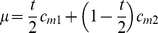
) but with an assumed 100% effectiveness. The women who experience unsuccessful medical abortion yet survive (proportion 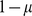) are also considered “lives saved”. Therefore the total number of lives saved for women who would have died from unsafe abortion is:
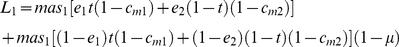
(1)The MMRatio is defined as number of maternal deaths per 100,000 live births in the population: 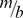
×100,000, where 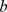 is total births/year. The adjusted MMRatio with the provision of medical abortion services is:
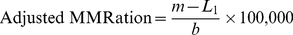
(2)


#### Additional potential medical abortion users

More women have unwanted pregnancies than attempt unsafe abortion. If women were confident that safe and effective induced abortion was available, more might induce abortion, including those who would otherwise have carried to term. These women would then face the mortality risks associated with medical abortion instead of those associated with giving birth and numbers of births would also be reduced. An extension to the original model estimates the additional number of lives that could be saved (see conceptual framework, Figure 2b).

To estimate additional medical abortions, number of births in each country, 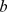, is multiplied by the proportion of births that we assume would have been aborted given the availability of medical abortion 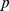, which we define as the proportion of women reporting their births as being unwanted or mistimed. Therefore additional demand for medical abortion is 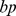. We assume medical abortion access is the same or lower for this group of women, 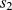, compared with those who would have undergone unsafe abortion. Therefore total numbers accessing medical abortion are 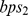. We assume that this group of women seek medical abortion during the first trimester in the same proportion as women who seek unsafe abortion (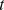). Number of lives saved depends on the mortality difference between carrying to term and undergoing medical abortion (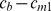,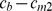), and the effectiveness of medical abortion (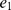,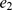). Therefore total deaths averted for women accessing medical abortion services who would otherwise have given birth is 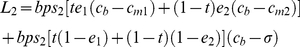
(3)where 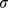 is analogous to the 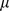 term in equation (1) and is defined depending on one of two scenarios: i) each woman carries to term and experiences the same risk of maternal death as other women giving birth in her country, 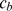; and ii) each woman seeks a repeat medical abortion (with assumed 100% effectiveness second time) with the mortality risk for medical abortion (same assumption regarding trimesters as Scenarios 2 and 4, 
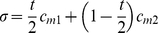
). There are no scenarios where women seek unsafe abortion because this group of women represents those who would have given birth rather than seek unsafe abortion. The total number of lives that could be saved through introducing medical abortion is therefore:
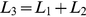
(4)The MMRatio now changes because number of births is substantially altered by providing medical abortion to women who would otherwise have given birth (we assume that the small change in births for Scenario 1, where women with failed medical abortions carry to term, has a negligible effect on the MMRatio and is ignored here). Births per year per country with medical abortion provision, 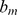, is defined as 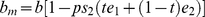 if we assume scenario i) or 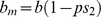 for scenario ii). The MMRatio is:
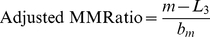
(5)


### Parameterization

Plausible parameter values and ranges were arrived at through a literature search (Table 1). A fuller description of parameter value justification is given in Supplementary Text S1.

**Table 1:** Model parameter descriptions and estimates, with sources.

Symbol	Parameter description		Tanzania	Ethiopia	Sources
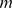	Maternal mortality (deaths/year)	High estimate	17,789	29,944	Middle estimates [39]; upper and lower estimates derived from 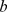 and MMRatio range.
		Middle estimate	13,000	22,000	
		Low estimate	8,484	14,056	
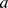	Proportion of maternal mortality that is abortion-related	Continental estimate	3.9%	3.9%	[6]
		Regional estimate	17%	17%	[2]
		Country estimate	21%	35%	[50], [51]
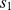 , 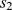	Proportion of women previously undergoing unsafe abortion ( 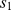 ) or previously giving birth ( 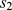 ) who have access to medical abortion.	Antenatal care coverage	95%	28%	[40], [52]
		Family planning services coverage	56%	31%	
		Primary healthcare coverage*	57%	19%	
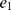 , 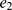	Medical abortion effectiveness	Misoprostol only: 1^st^ trimester	85% (73–92%)	85% (73–92%)	[15]–[26], [31], [53], [54]
		2^nd^ trimester	80% (65–85%)	80% (65–85%)	
		Mifepristone-misoprostol: both trimesters	96% (94–97%)	96% (94–97%)	
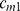 , 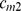	Mortality associated with medical abortion	1^st^ trimester	0.0001%‡	0.0001%‡	[55]
		2^nd^ trimester	0.0024%‡	0.0024%‡	
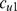 , 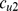	Mortality associated with unsafe abortion	1^st^ trimester	0.0852%‡	0.0852%‡	[2], [8]
		2^nd^ trimester	1.7032%‡	1.7032%‡	
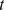	Proportion aborted pregnancies terminated in 1^st^ trimester		62% (60–85%)	62% (60–85%)	[56], [57]
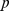	Proportion of pregnancies mistimed/unwanted		21.8%	33.8%	[40], [52]
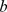	Total births/year		1,368,421†	3,055,556*	Derived from estimates of MMRatio and 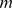 [39]
MMRatio	Maternal mortality ratio (maternal deaths/100,000 live births)		950 (620–1300)	720 (460–980)	[39]

UN – United Nations; UNFPA – United Nations Population Fund; UNICEF – United Nations Children's Fund; WHO – World Health Organization.

*Approximated by percentage of children <5 years for whom treatment was sought from a health facility or provider (excluding pharmacies, shops and traditional practitioners) for symptoms of acute respiratory infection.

† Figures for total births/year are in relatively good agreement to those derived from estimates of birth rate and population size for Tanzania and Ethiopia quoted in the CIA World Fact Book (derived estimates of 1,412,486 and 2,879,751 births/year for Tanzania and Ethiopia, respectively) [58].

‡ Calculated figures; see Supplementary Text S1 for details. Calculations are based on proportion of aborted pregnancies terminated in the first trimester, 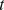, being 62%; where this value is varied in sensitivity analysis, these values are recalculated accordingly.

The proportions of women with access to medical abortion, 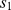 and 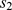, depend on the coverage achieved. To fully explore the potential of this intervention, the entire range of possible values (0–100% access) was investigated. Proportion of women accessing other health services such as antenatal care, family planning services and primary healthcare may indicate the possible coverage rates achievable for medical abortion if it were made available through these pre-existing health care service delivery networks (see Table 1). In the absence of empirical data, we assume 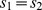 i.e. same access for all women, although unsafe abortion users may have higher access rates than other women (for example being more likely to live in urban areas).

## Results

**Figure 3:**
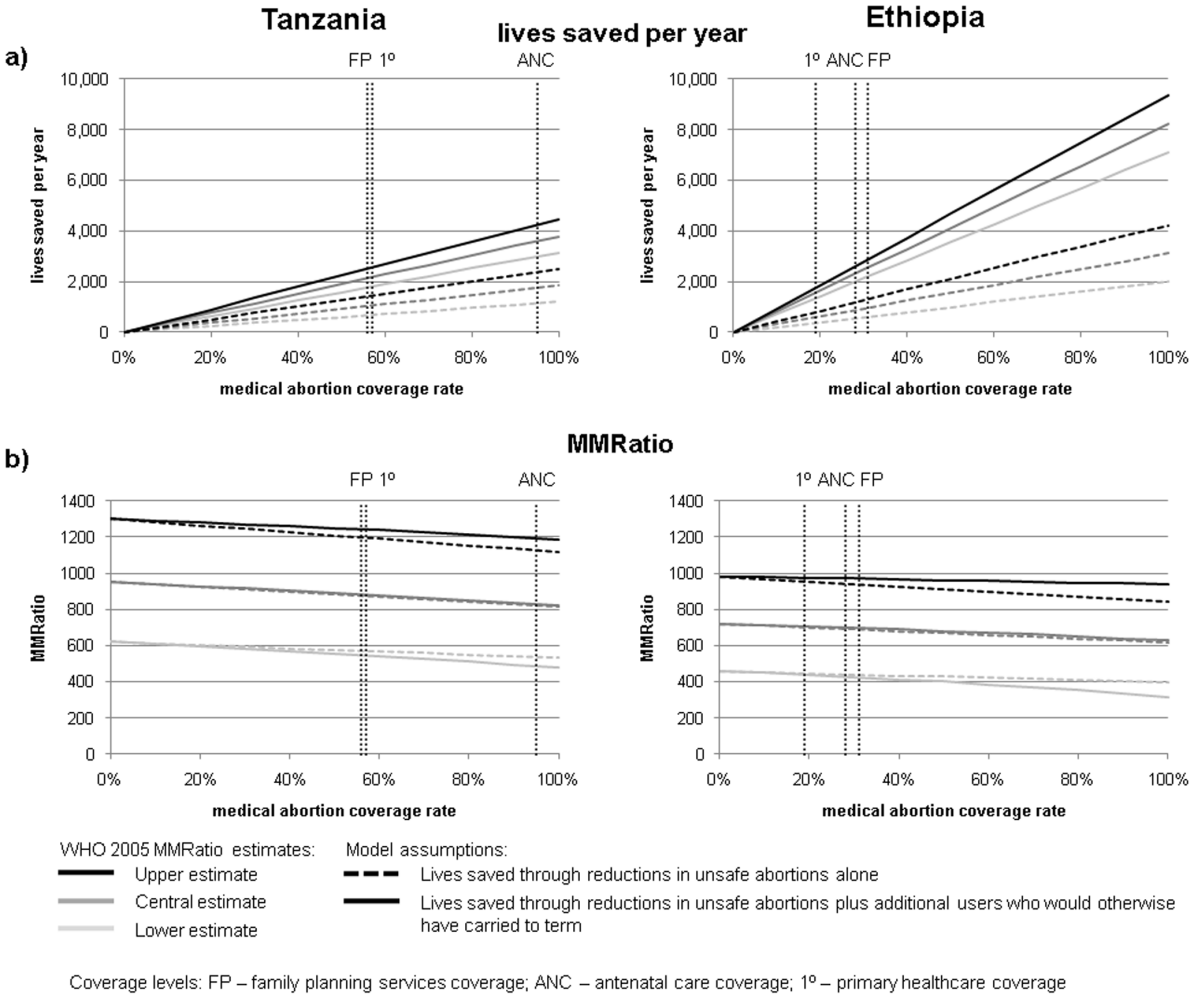
Relationship between hypothetical coverage level of medical abortion services and a) number of women's lives saved per year and b) the MMRatio for Tanzania and Ethiopia. All scenarios assumed medical abortion uses misoprostol-only (effectiveness 85% first trimester, 80% second trimester); that coverage of medical abortion services is the same for women who would have had an unsafe abortion and those who would have gone to term; proportion of maternal mortality that is abortion-related is 17% (regional estimate) and proportion of pregnancies which are unwanted/mistimed is 21.8% for Tanzania and 33.8% for Ethiopia. We assume a conservative estimate of lives saved by showing the worst case scenario: Figure 2a Scenario 3, Figure 2b Scenario i. That is, of those women whose medical abortion fails who would otherwise have died from unsafe abortion, 100% are assumed to die (from seeking a second, but unsafe, abortion). Women whose medical abortion fails who would otherwise have gone to term are assumed not to seek a second abortion of either type, and carry the same mortality risk associated with a pregnancy going to term.

Figure 3 illustrates the number of women's lives that could be saved by introducing medical abortion using our model. Feasible coverage scales are indicated on the figure. Because misoprostol-only may be a more feasible and accessible regimen for medical abortion in developing countries, primarily due to the cost of mifepristone, we chose to present results for a misoprostol-only regimen for our principle results.

Assuming that many unwanted/mistimed pregnancies that would have gone to term may be terminated through medical abortion saves thousands more women's lives per year than assuming an impact on unsafe abortion users only (1093 and 1587 more lives saved/year for Tanzania and Ethiopia respectively (2122 and 2551 saved in total), with coverage assumed at family planning service coverage levels: 56% and 31% respectively). However, these benefits are not reflected in the MMRatio reductions (assuming central MMRatio estimates, Table 1): an 8% reduction when only unsafe abortion users are considered compared to a 7% reduction when all potential users are included for Tanzania (4% and 3% figures for Ethiopia, respectively). The utilization of medical abortion services by women who would otherwise have carried to term decreases the birth rate; therefore the impact of lives saved on the MMRatio is reduced. The relationship between the MMRatio and coverage (Figure 3b) is not quite linear, yet considering all potential medical abortion users, each 10% increase in coverage broadly confers a 1.0–2.0% decrease in MMRatio for each country, saving 379 (Tanzania) and 823 (Ethiopia) women's lives.

### Model sensitivity

#### Choice of regimen

**Figure 4:**
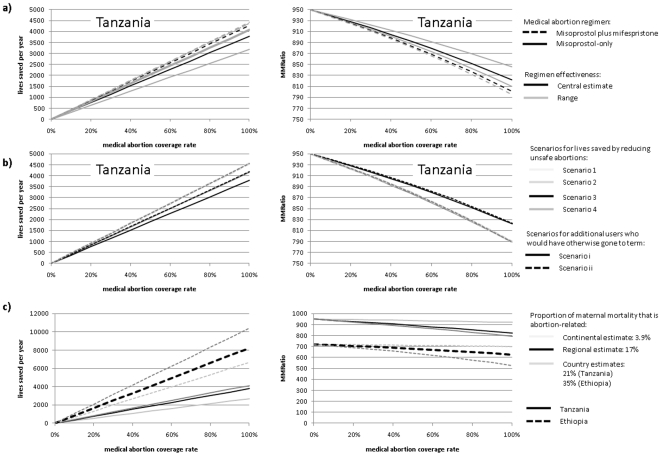
Sensitivity of model output (lives saved per year and MMRatio by medical abortion coverage rate) to different model assumptions and parameter values. a) Effectiveness of medical abortion regimen, for Tanzania: comparing misoprostol-only (estimated effectiveness 85% range 73–92% first trimester, 80% range 65–85% second trimester) with misoprostol plus mifepristone (estimated effectiveness 96% range 94–97%, both trimesters). b) Scenarios for those women who experience an unsuccessful medical abortion (see Figure 2 and Methods for descriptions of each scenario). c) Proportion of maternal mortality that is abortion-related, comparing national, regional and continental estimates. Results are for impact of medical abortion in terms of reduced unsafe abortions plus additional potential users. MMRatio graphs use central MMRatio estimate only. For a) and c) we assumed a conservative estimate of lives saved by showing the worst case scenario: Figure 2a Scenario 3, Figure 2b Scenario i.

The number of lives saved using misoprostol-only remains substantial (Figure 4a). For both Tanzania and Ethiopia, using central estimates for effectiveness of these regimens and assuming 100% access to medical abortion, misoprostol-only saved 13% fewer lives than mifepristone-misoprostol and mifepristone-misoprostol extends the reduction in MMRatio from 13% (misoprostol-only) to 16%.

#### Outcomes for women failing medical abortion

Within the model, women are subject to different outcomes depending on the scenarios adopted for failed medical abortions (as describe in the Methods). Sensitivity of lives saved and MMRatio estimates to these assumptions are shown in Figure 4b for Tanzania. Benefits of medical abortion are not sensitive to differences between scenarios 1, 2 and 4: these vary the future of women who would otherwise have had an unsafe abortion and died, and in these scenarios, the mortality risks for these women are *broadly* similar (that associated with giving birth, having an unsafe abortion or having a repeat medical abortion, respectively – even though unsafe abortion mortality risk is far higher than the other two, it is much lower than the 100% mortality of scenario 3). In contrast, scenario 3 assumes *all* these women would die as a result of unsafe abortion, and so mitigates the impact of medical abortion quite substantially. Benefits are also sensitive to the future of women who would otherwise have carried to term who have a failed medical abortion (scenarios i and ii), primarily because this is such a large group of users.

#### Proportion of maternal mortality that is abortion-related

Benefits are also highly sensitive to estimates of this assumption (Figure 4c), highlighting the importance of collecting cause-specific maternal mortality data at a national level, despite the methodological challenges.

## Discussion

Thousands of lives could be saved by introducing medical abortion in Ethiopia and Tanzania, reducing their national MMRatios. Universal medical abortion coverage reduces MMRatios by 13% from current estimates for both countries, with each 10% increase in coverage broadly conferring a 1.0–2.0% decrease. This constitutes considerable progress towards MDG 5. The political climate towards abortion in Africa is showing signs of reform, with Ethiopia recently having relaxed restrictions on abortion provision and Ghana having approved national standards and guidelines for safe abortion that include medical abortion. The Obama administration's reversal of the “global gag rule” now permits federal funding to be awarded to organisations that offer abortions. Availability of misoprostol has also increased with its registration in several African countries for use in many obstetrics and gynaecology indications. Therefore access to medical abortion looks set to increase substantially.

To our knowledge this is the first model to explore the wider social impact of introducing legal medical abortion, by examining its uptake by women who would otherwise have carried to term, as well as women who would have undergone unsafe abortion. In countries where a high proportion of maternal mortality is due to unsafe abortion, the reduction in MMRatio could be dramatic if sufficient coverage could be achieved. If the higher estimates for Ethiopia's abortion-attributable-maternal mortality are correct (35%), the universal availability of misoprostol could reduce Ethiopia's MMRatio from 720 to 528, giving it a lower MMRatio than Bangladesh, a country 32 places above Ethiopia on the Human Development Index [39]. Even assuming low abortion-attributable-maternal mortality (3.9%), Ethiopia could save upwards of 6500 lives annually with universal access.

We found that substantial numbers of women's lives saved by provision of medical abortion was not always reflected in great reductions in MMRatios, because if women who would otherwise have given birth use these services, the birth rate may alter considerably. The reduced birth rate implied by our model is likely an overestimate, as many women with mistimed pregnancies who terminate may have additional, future pregnancies, and not all women with unwanted/mistimed pregnancies would necessarily seek a medical abortion. However our analysis highlights that the success of medical abortion in reducing numbers of maternal deaths may not be adequately reflected in MMRatio statistics.

The area of greatest uncertainty in our simulation is the likely coverage of medical abortion services in each country, were it to be legalized (and therefore we have presented figures displaying medical abortion benefits using a continuous scale of coverage). An unwanted or mistimed pregnancy does not necessarily imply that a woman would terminate given the availability of medical abortion services. It is difficult to anticipate how introduction of legal abortion services might affect abortion-seeking behaviour in these settings. The use of family planning as a proxy for access to medical abortion could be problematic as one would assume that women with unwanted pregnancies are the same women without access to contraception. We would argue that the barriers and motivations involved in contraceptive use are significantly different from that of abortion. Access to contraception must be continuous, motivation to contracept must be sustained, and side effects such as changes to the menstrual cycle must be tolerated over time. However, with abortion access is “one-off”, motivation is heightened and one could argue that the gravity of an unwanted pregnancy for many women would mean that potential side effects would be more tolerable. The potential for medical abortion to be distributed at antenatal clinics by midwives may mean that up to 95% of Tanzanian women could access medical abortion [40]. However, Ethiopia has much weaker healthcare systems and therefore alternative means for distribution and availability should be explored.

Coverage will also depend on the proportion of health care workers willing to provide medical abortion. It is difficult to anticipate this percentage because there is not much research on this topic in Africa [41]. We found studies of provider support that ranged from 6% to 80%. Research in KwaZulu Natal, South Africa in 2000 showed that only 6% of nurses supported abortion on request (18% and 6%, respectively). However, a clear hierarchy of support was observed: a majority of nurses (56%) supported abortion in the case of rape or incest, or if the continued pregnancy would endanger a woman's health (61%, respectively), but few supported abortion for social or economic reasons [42]. However, research in South Africa has shown that it is possible to change attitudes, with 70% of respondents reporting behavioural changes six months after the workshop and 93% reporting increased compassion for women seeking abortion services [43]. In Ghana 80%, of physicians favoured the establishment of safe abortion units within national health facilities. Of these, 36% were willing to take part in counselling only, 45% were prepared to carry out abortions, and 19% said they would play no role in these units [44]. There is anecdotal evidence that providers prefer medical abortion because the women take the tablets themselves and thus they, rather than the provider, induce the abortion.

The number of women able to obtain a successful medical abortion is also contingent upon timely access. Educating women on how to detect a pregnancy early and where to access appropriate services quickly will be key to maximizing the impact of this intervention. Medical abortion services could be socially marketed as is done with other commodities such as contraceptives, bednets and oral rehydration salts, but the political sensitivity in many countries to abortion services makes this unlikely.

There are many limitations to our analysis. For simplicity, the model does not include spontaneous abortion and we assumed that a second medical abortion after a first (failed) procedure would be 100% successful. We assumed a low mortality for medical abortion based on data from the United States, but this rate (and rate of complications such as uterine rupture) may increase within developing countries if women take misoprostol in larger doses or late in pregnancy [45], or if they could not access post-abortion care. The lack of available data on the prevalence of these potential complications in a developing country setting demonstrates the need for further research.

Our analysis involved various assumptions regarding outcomes after unsuccessful medical abortions, explored through scenario analysis. Success rates could be improved through the implementation of effective protocols which administer second doses of misoprostol or mifepristone-misoprostol regimens, or refer women to hospitals for surgical procedures.

Additional users in the form of women who give birth but feel their pregnancy was unwanted/mistimed is likely overestimated, as not all these women would necessarily seek a medical abortion. A further group of additional potential users that are excluded for simplicity are women who would have undergone an unsafe abortion but survived, who would preferentially use medical abortion services. Data are lacking on total numbers accessing unsafe abortions in these settings: while an estimated 19 million unsafe abortions are performed annually in sub-Saharan Africa [7], there is considerable uncertainty surrounding this estimate. Furthermore the mortality risk of medical abortion is low compared with that for unsafe abortion, so additional numbers of deaths attributable to medical abortion from this group would be low; however uptake of medical abortion by this group would likely have a considerable impact on maternal morbidity.

Our analysis is limited to predicting the impact of a single intervention in reducing maternal mortality, but there are alternatives, such as increasing access to different termination methods, including manual vacuum aspiration. In particular, expanding access to family planning services would circumvent the need for abortion services in many cases. Family planning combined with safe abortion provision has been predicted to be the most cost-effective method of reducing maternal mortality in Mexico [33]. We can also predict that family planning would lead to a substantial reduction in maternal mortality, since the most lives saved through medical abortion provision predicted by our model was among women with unwanted pregnancies who would otherwise have continued to term.

While unsafe abortion is ranked as the fourth most common cause of maternal mortality worldwide, it is listed as the number one cause of maternal morbidity [46], with estimates suggesting 78 cases of temporary or permanent disability for every unsafe abortion-related death [2], [3]. Unsafe abortion has been linked to an increased risk of ectopic pregnancy, premature delivery, and spontaneous abortions in subsequent pregnancies [4]. The full impact of unsafe abortion is therefore not limited to the number of directly attributable deaths. We did not evaluate the disability-adjusted life years that could be saved by making medical abortion available due to lack of data. More research is required to assess the absolute costs of unsafe abortion because introducing medical abortion is likely to have positive effects reaching far beyond mortality statistics. It is widely recognized that interventions providing safe abortion services decrease costs to the health system through reducing complications from unsafe abortion [34], [47]. In some countries as many as two in three maternity beds are taken up by women hospitalized for treatment after an unsafe abortion and up to half of all ob-gyn budgets can be spent on this problem alone [4]. In Tanzania the cost of treating a woman with complications from unsafe abortion is over seven times the overall Ministry of Health budget per head of the population [4]. Medical abortion can serve only to increase cost effectiveness as expenditure on clinical personnel and facilities can be minimized.

Introducing medical abortion in countries with high abortion-related mortality is a feasible step towards achieving MDG 5, yet interventions that tackle other causes of maternal mortality must not be neglected [48]. However, many of these interventions require substantial funding and resources. Medical abortion does not require skilled health workers for administration, can be easily provided outside health centers, and is portable and easily stored meaning it can reach even remote rural areas. Several studies have demonstrated that mid-level providers can provide abortion services, including the diagnosis of early pregnancy and ectopic awareness safely and effectively [49]. While safe abortion services in any form would save women's lives, medical abortion offers resource-poor countries the tools to achieve this with minimal expenditure.

## Supporting Information

Text S1 (0.09 MB DOCX)

## References

[1] United Nations Website: Millennium Development Goals.. http://www.un.org/millenniumgoals/.

[2] World Health Organisation (2004). Unsafe Abortion: Global and Regional Estimates of the Incidence of Unsafe Abortion and Associated Mortality in 2000, 4th Edition.. http://whqlibdoc.who.int/publications/2004/9241591803.pdf.

[3] World Bank (2007). HNP Discussion Paper. Population Issues in the 21st Century: The Role of the World Bank.. http://siteresources.worldbank.org/HEALTHNUTRITIONANDPOPULATION/Resources/281627-1095698140167/PopulationDiscussionPaperApril07Final.pdf.

[4] Grimes DA, Benson J, Singh S, Romero M, Ganatra B (2006). Unsafe abortion: the preventable pandemic.. Lancet.

[5] United Nations. The Millennium Development Goals Report. New York 2008.. http://www.un.org/millenniumgoals/.

[6] Khan KS, Wojdyla D, Say L, Gulmezoglu AM, Van Look PF (2006). WHO analysis of causes of maternal death: a systematic review.. Lancet.

[7] Warriner I ‘Unsafe Abortion: An Overview of Priorities and Needs’..

[8] The Alan Guttmacher Institute (1999). Sharing Responsibility: Women, Society and Abortion Worldwide. New York.. http://www.guttmacher.org/pubs/sharing.pdf.

[9] Henshaw SK, Singh S, Haas T (1999). The incidence of abortion worldwide.. Int Fam Plann Persp.

[10] Creinin MD (2000). Medical abortion regimens: historical context and overview.. Am J Obstet Gynecol.

[11] Grossman D, Blanchard K, Blumenthal P (2008). Complications after second trimester surgical and medical abortion.. Reprod Health Matters.

[12] Willmott FJ, Scherf C, Ford SM, Lim K (2008). Rupture of uterus in the first trimester during medical termination of pregnancy for exomphalos using mifepristone/misoprostol.. BJOG.

[13] Goyal V (2009). Uterine rupture in second-trimester misoprostol-induced abortion after cesarean delivery: a systematic review.. Obstet Gynecol.

[14] Majoko F, Magwali T, Zwizwai M (2002). Uterine rupture associated with use of misoprostol for induction of labor.. Int J Gynaecol Obstet.

[15] Kahn JG, Becker BJ, MacIsaa L, Amory JK, Neuhaus J (2000). The efficacy of medical abortion: a meta-analysis.. Contraception.

[16] von Hertzen H, Piaggio G, Huong NT, Arustamyan K, Cabezas E (2007). Efficacy of two intervals and two routes of administration of misoprostol for termination of early pregnancy: a randomised controlled equivalence trial.. Lancet.

[17] Bhattacharjee N, Saha SP, Ghoshroy SC, Bhowmik S, Barui G (2008). A randomised comparative study on sublingual versus vaginal administration of misoprostol for termination of pregnancy between 13 to 20 weeks.. Aust N Z J Obstet Gynaecol.

[18] Carbonell JL, Rodriguez J, Aragon S, Velazco A, Tanda R (2001). Vaginal misoprostol 1000 microg for early abortion.. Contraception.

[19] Carbonell JL, Varela L, Velazco A, Fernandez C (1997). The use of misoprostol for termination of early pregnancy.. Contraception.

[20] Coyaji K, Elul B, Krishna U, Otiv S, Ambardekar S (2002). Mifepristone-misoprostol abortion: a trial in rural and urban Maharashtra, India.. Contraception.

[21] Hajri S (2004). Medical abortion: the Tunisian experience.. Afr J Reprod Health.

[22] Hamoda H, Ashok PW, Flett GM, Templeton A (2005). Medical abortion at 9–13 weeks' gestation: a review of 1076 consecutive cases.. Contraception.

[23] Kapp N, Borgatta L, Stubblefield P, Vragovic O, Moreno N (2007). Mifepristone in second-trimester medical abortion: a randomized controlled trial.. Obstet Gynecol.

[24] Mundle S, Elul B, Anand A, Kalyanwala S, Ughade S (2007). Increasing access to safe abortion services in rural India: experiences with medical abortion in a primary health center.. Contraception.

[25] Thi Nhu Ngoc N, Winikoff B, Clark S, Ellertson C, Ngoc Am K (1999). Safety, Efficacy and Acceptability of Mifepristone-Misoprostol Medical Abortion in Vietnam.. International Family Planning Perspectives.

[26] Winikoff B, Sivin I, Coyaji KJ, Cabezas E, Bilian X (1997). The Acceptability of Medical Abortion in China, Cuba, and India.. International Family Planning Perspectives.

[27] Miller S, Lehman T, Campbell M, Hemmerling A, Anderson SB (2005). Misoprostol and declining abortion-related morbidity in Santo Domingo, Dominican Republic: a temporal association.. BJOG.

[28] Barbosa RM, Arilha M (1993). The Brazilian experience with Cytotec.. Stud Fam Plann.

[29] Berer M (2005). Medical abortion: a fact sheet.. Reprod Health Matters.

[30] Costa SH, Vessey MP (1993). Misoprostol and illegal abortion in Rio de Janeiro, Brazil.. Lancet.

[31] Harper CC, Blanchard K, Grossman D, Henderson JT, Darney PD (2007). Reducing maternal mortality due to elective abortion: Potential impact of misoprostol in low-resource settings.. Int J Gynaecol Obstet.

[32] Prata N, Sreenivas A, Vahidnia F, Potts M (2009). Saving maternal lives in resource-poor settings: facing reality.. Health Policy.

[33] Hu D, Bertozzi SM, Gakidou E, Sweet S, Goldie SJ (2007). The costs, benefits, and cost-effectiveness of interventions to reduce maternal morbidity and mortality in Mexico.. PLoS ONE.

[34] Hu D, Grossman D, Levin C, Blanchard K, Goldie SJ (2009). Cost-effectiveness analysis of alternative first-trimester pregnancy termination strategies in Mexico City.. BJOG.

[35] Jowett M (2000). Safe Motherhood interventions in low-income countries: an economic justification and evidence of cost effectiveness.. Health Policy.

[36] Bhutta ZA, Ali S, Cousens S, Ali TM, Haider BA (2008). Alma-Ata: Rebirth and Revision 6 Interventions to address maternal, newborn, and child survival: what difference can integrated primary health care strategies make?. Lancet.

[37] Bartlett LA, Berg CJ, Shulman HB, Zane SB, Green CA (2004). Risk factors for legal induced abortion-related mortality in the United States.. Obstet Gynecol.

[38] Fernandez MM, Coeytaux F, de Leon RGP, Harrison DL (2009). Assessing the global availability of misoprostol.. International Journal of Gynecology & Obstetrics.

[39] World Health Organisation, Maternal Mortality in 2005: Estimates developed by WHO, UNICEF, UNFPA and The World Bank.. http://www.who.int/making_pregnancy_safer/documents/9789241596213/en/index.html.

[40] National Bureau of Statistics (NBS) [Tanzania] and ORC Macro (2005). Tanzania Demographic and Health Survey 2004–05.

[41] Harries J, Stinson K, Orner P (2009). Health care providers' attitudes towards termination of pregnancy: a qualitative study in South Africa.. BMC Public Health.

[42] Harrison A, Montgomery ET, Lurie M, Wilkinson D (2000). Barriers to implementing South Africa's Termination of Pregnancy Act in rural KwaZulu/Natal.. Health Policy Plan.

[43] Turner KL, Hyman AG, Gabriel MC (2008). Clarifying values and transforming attitudes to improve access to second trimester abortion.. Reprod Health Matters.

[44] Morhe ES, Morhe RA, Danso KA (2007). Attitudes of doctors toward establishing safe abortion units in Ghana.. Int J Gynaecol Obstet.

[45] Lovold A, Stanton C, Armbruster D (2008). How to avoid iatrogenic morbidity and mortality while increasing availability of oxytocin and misoprostol for PPH prevention?. Int J Gynaecol Obstet.

[46] World Health Organisation Mother-Baby Package (1994). Implementing safe motherhood in countries.. http://whqlibdoc.who.int/hq/1994/WHO_FHE_MSM_94.11_Rev.1.pdf.

[47] Levin C, Grossman D, Berdichevsky K, Diaz C, Aracena B (2009). Exploring the costs and economic consequences of unsafe abortion in Mexico City before legalisation.. Reprod Health Matters.

[48] Ronsmans C, Graham WJ (2006). Maternal mortality: who, when, where, and why.. Lancet.

[49] Berer M (2009). Provision of abortion by mid-level providers: international policy, practice and perspectives.. Bull World Health Organ.

[50] Gebreselassie H, Fetters T (2002). Responding to unsafe abortion in Ethiopia: A facility-based assessment of postabortion care services in public-health sector facilities in Ethiopia.. http://www.ipas.org/Publications/asset_upload_file965_2470.pdf.

[51] United Nations Population Division (No date). Abortion policies: a global review.. http://www.un.org/esa/population/publications/abortion/doc/tanzania.doc.

[52] Central Statistical Agency [Ethiopia] and ORC Macro (2006). Ethiopia Demographic and Health Survey 2005.. http://www.measuredhs.com/pubs/pdf/FR179/FR179.pdf.

[53] Billings DL (2004). Misoprostol alone for early medical abortion in a Latin American clinic setting.. Reprod Health Matters.

[54] Jain JK, Dutton C, Harwood B, Meckstroth KR, Mishell DR (2002). A prospective randomized, double-blinded, placebo-controlled trial comparing mifepristone and vaginal misoprostol to vaginal misoprostol alone for elective termination of early pregnancy.. Hum Reprod.

[55] Grimes DA (2005). Risks of mifepristone abortion in context.. Contraception.

[56] Gebreselassie H, Fetters T, Singh S, Abdella A, Gebrehiwot Y (2010). Caring for women with abortion complications in Ethiopia: national estimates and future implications.. Int Perspect Sex Reprod Health.

[57] Bankole A, Sedgh G, Oye-Adeniran BA, Adewole IF, Hussain R (2008). Abortion-seeking behaviour among Nigerian women.. J Biosoc Sci.

[58] United Nations, Statistics, Demographic Yearbook.. https://www.cia.gov/library/publications/the-world-factbook/.

