# Supplementary material for: The Potential of Medical Abortion to Reduce Maternal Mortality in Africa: What Benefits for Tanzania and Ethiopia?
Source: PLoS One. 2010 Oct 11;5(10):e13260. doi: 10.1371/journal.pone.0013260 (PMC2952582; doi:10.1371/journal.pone.0013260)
Supplement: Text S1 — (0.09 MB DOCX) [file pone.0013260.s001.docx]

**Supplementary Text S1**

**Exploring the Potential of Medical Abortion to Reduce Maternal Mortality in Africa: What benefits for Tanzania and Ethiopia?**

*Baggaley et al.*

**Parameterization**

*Maternal mortality,* $m$

The maternal mortality for each country is taken from established estimates for maternal mortality developed by WHO, UNICEF, and UNFPA [60]. The WHO only provides regional estimates of the proportion of maternal deaths attributable to unsafe abortion, $a$, and this does not account for variation between countries. National estimates were therefore obtained from results reported from stand-alone studies; all stand-alone studies reported significantly higher abortion-related mortality than WHO regional statistics [3,51,52,61,62].

*Effectiveness,* $e_{1}$,$e_{2}$

The success rate, that is, effectiveness of medical abortion, $e_{1}$ and $e_{2}$ for first and second trimester abortions respectively, were obtained from an extensive search of the literature. Effectiveness appears to decrease with increasing gestation for misoprostol-only but not significantly for misoprostol-mifepristone regimens [16,18,19,20,21,22,23,24,25,26,27].

There are almost no available data on the use of medical abortion in developing countries after nine weeks. The efficacy rates quoted in the studies used to derive our overall efficacy for mifepristone-misoprostol and misoprostol-only regimens have not been demonstrated in a developing country setting and it is therefore possible we may have overestimated the regimens’ potential success, although we have aimed to keep our estimates conservative. A previous modeling analysis by Harper et al assumed misoprostol-only effectiveness to be 90% for first trimester and 85% for second trimester [32]. We assume a more conservative 85% estimate for first trimester medical abortions based on recent data [17] and use the same 6% reduction in effectiveness of Harper et al, thus assuming 80% effectiveness for the second trimester.

*Proportion of births unwanted or mistimed,* $p$

To estimate additional potential users of medical abortion, a proxy was required to estimate the proportion of births that would have been aborted if medical abortion was available. It has been estimated that of all births in Africa, 8% are unwanted and 9% are mistimed [10]. Therefore we assume$p$is 17% to demonstrate the maximum potential of providing medical abortion and in recognition that an underreporting bias may exist in women stating that their children were unwanted or mistimed.

*Proportion of maternal mortality attributable to unsafe abortion,* $a$

Estimates for the proportion of maternal mortality attributable to abortion must be viewed with caution. WHO figures give only a regional estimate for East Africa. Within this region the proportion of abortion-related maternal mortality will likely vary country by country. A short literature search indicates that Ethiopia’s maternal mortality is greatly influenced by unsafe abortion procedures. There have been many stand-alone studies conducted on the proportion of maternal mortality attributable to abortion in Ethiopia, mostly in urban settings from hospital records. Estimates of 27%, 35%, 52% and 55% of maternal mortality being abortion-related were found in the literature [61,63]. The estimate of 35% was chosen to illustrate the higher abortion-related mortality while still exercising caution.

Due to the lack of country-specific data on abortion-related mortality and unwanted/mistimed births, the parameters employed for both these variables were regional, for East Africa and Africa respectively. Indeed, the very nature of abortion makes it difficult to obtain reliable statistics and therefore even country-specific estimates must be employed with caution.

Mortality associated with medical (*(* $c_{m1}$,$c_{m2}$) and unsafe ($c_{u1}$,$c_{u2}$) abortion

Mortality has been estimated as 0.001% [56] and 0.7% [3,10] for medical and unsafe abortion respectively, but there is a lack of information on how these rates are affected by gestational age. We therefore used information on risk from surgical abortion, where in the US it was found that the unadjusted relative risk of abortion-related mortality was 1.4 (95% confidence interval 0.5-4.2) at 9-10 weeks gestation, 3.4 (1.2-9.7) at 11-12 weeks, 14.7 (6.2-34.7) at 13-15 weeks, 29.5 (12.9-67.4) at 16-20 weeks and 76.6 (32.5-180.8) at ≥21 weeks, compared with abortion at ≤8 weeks [38]. From this, we approximated that medical and unsafe abortion would confer a 20-fold increased mortality risk if performed in the second trimester. The average mortality risk for medical abortion across both trimesters can be represented as $c_{\bar{m}}=c_{m1}t+c_{m2}\left(1-t\right)$ and for unsafe abortion as $c_{\bar{u}}=c_{u1}t+c_{u2}\left(1-t\right)$. Therefore the risks during the first trimester are $c_{m1}=\frac{c_{\bar{m}}}{t+v\left(1-t\right)}$ and $c_{u1}=\frac{c_{\bar{u}}}{t+v\left(1-t\right)}$ respectively, where $v$ represents the -fold increased mortality risk by performing the procedure in the second trimester (estimated as 20-fold). Table 1 gives values for $c_{m1}$,$c_{m2}$,$c_{u1}$,$c_{u2}$ assuming that the proportion terminating in the first trimester, $t$, is 62% and these are the values used in all analyses except where $t$ is varied in the sensitivity analysis, in which case these mortality risks are adjusted accordingly.

*Access,* $s_{1}$,$s_{2}$

The access parameters were obtained from World Bank statistics and Demographic Health Surveys [64] and are based on antenatal coverage, percentage demand for family planning that is met, and percent of children with ARI accessing health facilities: all services which may be seen to possess similar qualities to those needed for medical abortion distribution. These data are widely used and considered more reliable than smaller stand-alone studies.

*Proportion of aborted pregnancies terminated in the first trimester,* $t$

A recent nationally representation sample of 1,932 women in Ethiopia seeking postabortive care in public and private health facilities estimated that 62% of abortions were in the first trimester, with the remainder in the second. We found no such estimates for Tanzania, but found estimates of 80.6% of women in Nigeria [58] and 88.0% in the United States [65] aborting at <12 weeks gestation. We assumed that access to abortion services would be more difficult in African settings than in the US and so varied the proportion between 60% and 85% for the sensitivity analysis, using a fixed estimate of 62% for the other analyses. Family planning services and primary healthcare coverage are higher in Tanzania than Ethiopia (Table 1) and so the proportion terminating at earlier gestations may well be higher than 62% in Tanzania, but in the absence of any data to support this, we elected to keep the proportions for the two countries the same.
